# TikTok as a Platform for Patient Education and Health Information in Rare Genetic Diseases: Cross-Sectional Study

**DOI:** 10.2196/79978

**Published:** 2026-02-24

**Authors:** Jackson Montgomery Wahman, Rhoda Mariam Hijazi, Hosam Gharib Abdelhady

**Affiliations:** 1 College of Osteopathic Medicine, Sam Houston State University, Conroe, TX, United States; 2 Department of Physiology and Pharmacology, College of Osteopathic Medicine, Sam Houston State University, 925 City Central Ave, Conroe, TX, United States, 1 936-202-5221

**Keywords:** rare diseases, social media, patient education, health misinformation, TikTok, Ehlers-Danlos syndrome, Marfan syndrome, cystic fibrosis, Gaucher disease

## Abstract

**Background:**

Rare genetic diseases pose significant diagnostic and therapeutic challenges, often leading to delayed diagnoses, misinformation, and patient isolation. Social media platforms have emerged as prominent spaces for health information dissemination and community building among patients with rare diseases.

**Objective:**

This study aimed to evaluate the role of TikTok videos in patient education, community engagement, and information quality related to 5 rare genetic conditions: Ehlers-Danlos syndrome, Marfan syndrome, cystic fibrosis, Wilson disease, and Gaucher disease.

**Methods:**

A cross-sectional analysis was conducted on 184 TikTok videos identified via disease-specific hashtags. Included videos were 15 seconds to 4 minutes long and directly discussed the target diseases. Advertisements, promotional content, and product marketing were excluded. Videos were categorized by creator type: physicians, medical professionals, patients, influencers, nonprofit organizations, and others. Content quality was assessed using the Global Quality Scale (GQS) and a modified DISCERN tool (mDISCERN). Engagement metrics (views, likes, and shares) were recorded. Kruskal-Wallis and chi-square tests evaluated differences across creator categories.

**Results:**

Of the 184 TikTok videos, 88 (47.8%) were created by patients or family members; 31 (16.8%) by influencers, 24 (13.0%) by physicians, 17 (9.2%) by nonprofit organizations, 15 (8.2%) by general users, and 9 (4.9%) by others. Collectively, the videos amassed more than 123 million views. Influencer-generated content accounted for the highest cumulative view count, totaling approximately 60.9 million views. Content produced by medical professionals and physicians demonstrated higher information quality, with mean GQS scores of 3.89 (SD 0.66) and 3.62 (SD 0.71) and mDISCERN scores of 3.11 (SD 0.58) and 3.21 (SD 0.65), respectively. In contrast, videos by influencers and patients exhibited lower quality scores (influencers: GQS mean 1.48, SD 0.60; mDISCERN mean 1.42, SD 0.55; patients: GQS mean 1.57, SD 0.58; mDISCERN mean 1.38, SD 0.52). For Ehlers-Danlos syndrome (n=40 videos, 21.7%), Wilson disease (n=40 videos, 21.7%), and cystic fibrosis (n=34 videos, 18.5%), significant differences in quality scores among creator types were observed (*P*<.001, *P*<.001, and *P*≤.04, respectively). For Marfan syndrome (n=40 videos, 21.7%) and Gaucher disease (n=30 videos, 16.3%), no significant differences were observed (*P*=.43 and *P*=.07, respectively). Chi-square analysis indicated no association between creator type and inclusion of peer-reviewed references (*χ*^2^_5_=10.6; *P*=.07). Overall, only 7 (3.8%) videos cited scientific literature.

**Conclusions:**

TikTok serves as a key platform for rare disease awareness and community engagement, although the quality and accuracy of health information vary widely. Although medical professionals produced higher-quality content, it tended to receive less visibility. Increasing the presence of health care professionals and improving visibility of evidence-based content could enhance patient education and safer health information sharing.

## Introduction

Rare genetic conditions such as Ehlers-Danlos syndrome (EDS), Marfan syndrome, and Wilson disease often present with nonspecific or variable symptoms. These characteristics contribute to diagnostic delays, fragmented care, and social isolation for affected individuals [1,2]. This aligns with broader evidence that individuals with rare disorders often face significant gaps in health literacy, limited access to information, and barriers to effective self-management, which underscores the importance of peer support and alternative educational tools [3]. In the absence of robust institutional resources or consistent health care professional familiarity, many patients and caregivers turn to social media for support, disease education, and practical guidance [4,5]. These 5 rare genetic conditions (EDS, Marfan syndrome, cystic fibrosis, Wilson disease, and Gaucher disease) were selected to capture diversity in etiology (connective tissue, metabolic, and pulmonary) and representation on TikTok. Each condition has a distinct clinical profile and active online patient community, which facilitates consistent comparison of creator engagement and educational content across diseases [6-8].

Among social media platforms, TikTok has emerged as a dominant venue for health discourse. With over 1 billion monthly users globally and strong penetration among adolescents and young adults, TikTok is uniquely positioned to influence health knowledge, particularly in underrepresented or rare conditions [6]. The platform’s algorithmic design enables rapid exposure to highly personalized video content, often delivered without intentional searching. This makes TikTok a powerful yet potentially unreliable source of health information [7].

Despite TikTok’s popularity, little research has specifically examined the accuracy and educational impact of rare disease–related content, a gap this study aims to address. In rare disease communities, TikTok fosters connection and visibility. Patients and families use hashtags such as #RareDisease and #ChronicIllness to share lived experiences, raise awareness, and advocate for research and support services [8]. Video content frequently documents diagnostic journeys, daily management, and emotional coping. These narratives contribute to psychosocial support and patient empowerment [8,9]. However, the ease of content creation also raises concerns about the quality and accuracy of medical information that reaches potentially vulnerable audiences [10,11].

Recent studies across several health domains reveal that TikTok videos commonly lack references to scientific literature and often include misinformation, especially those created by influencers or nonmedical users [12,13]. For example, a review of prostate cancer–related videos on TikTok found that nearly half of informational videos contained false or misleading statements [14]. Similar trends have been observed in videos addressing attention-deficit/hyperactivity disorder (ADHD), fibromyalgia, sinusitis, and eating disorders, where professional content consistently surpasses user-generated videos in quality [15,16].

Validated tools such as the Global Quality Scale (GQS) and DISCERN have been used to assess the reliability and usefulness of online medical content, including short-form videos [11,17]. Although originally developed for written or long-form materials, these tools are now applied to TikTok videos to enable comparisons across different content creators [18,19]. To address their known limitations in short-form contexts, the modified DISCERN (mDISCERN) version, adapted for audiovisual and short-form media in prior research, was used in this study alongside GQS to maintain cross-study comparability while improving contextual relevance [18].

This study evaluated the information quality and audience reach of TikTok videos related to 5 rare genetic conditions: EDS, Marfan syndrome, cystic fibrosis, Wilson disease, and Gaucher disease. Using established quality measures, we examined whether content produced by medical professionals differs from that produced by patients, influencers, and general users. We also analyzed how these differences correspond to viewer engagement. Through this approach, we aimed to highlight both the educational potential and the limitations of TikTok as a platform for rare disease communication.

## Methods

### Overview

This study used a cross-sectional observational design to evaluate TikTok videos discussing 5 rare genetic conditions: EDS, Marfan syndrome, cystic fibrosis, Wilson disease, and Gaucher disease. Videos were identified using TikTok’s native search function within the mobile app. Raters created new TikTok accounts to avoid the influence of personalized algorithms. For each condition, standardized English-language hashtags were entered into the search bar and manually screened in descending order of view count as displayed by the platform. The following search terms were used:

- For EDS, #EhlersDanlos, #EDS, #EhlersDanlosSyndrome
- For Marfan syndrome, #Marfan, #MarfanSyndrome
- For cystic fibrosis, #CysticFibrosis, #CFWarrior, #CFCommunity
- For Wilson disease, #WilsonsDisease, #WilsonDisease, #CopperToxicity
- For Gaucher disease, #GaucherDisease, #Gaucher

The first 40 videos with the highest visible view counts were included for eligibility screening, yielding 200 total videos. Videos were eligible if they were posted between January 2022 and October 2024, directly discussed the target condition, and were between 15 seconds and 4 minutes in duration, consistent with the length range of short-form health content on TikTok reported in prior studies [17]. Exclusion criteria included advertisements, promotional content, duplicate uploads, and non-English videos. After exclusions, 184 unique videos remained for analysis. All searches were conducted in December 2024 to ensure that the dataset reflected the most current content available at the time of analysis. The inclusion period (January 2022 to October 2024) was selected because TikTok expanded its maximum video length from 50 seconds to 10 minutes in early 2022, allowing for more substantive educational content. The cutoff of October 2024 was chosen to include approximately 3 years of postexpansion content while maintaining a manageable and temporally consistent dataset.

Videos were categorized by creator type based on account biographies, stated credentials, and linked websites. Categories included physicians, medical professionals, patients, influencers, nonprofit organizations, and others. Creators were classified as physicians if their profile or linked page listed an MD, DO, or an international equivalent medical degree (eg, MBBS). Medical professionals included other licensed health care workers such as registered nurses, genetic counselors, pharmacists, or physical therapists who reported professional credentials but did not hold physician degrees. This distinction was made to explore whether engagement and information quality differed between physician-led and allied health–led educational content, reflecting the unique training and communication roles of each group in digital health spaces. Influencers were defined as creators with a substantial follower base (typically exceeding 10,000 followers) or self-identified content creators who regularly produced health-related or lifestyle videos for a broad public audience but did not hold formal medical credentials or represent nonprofit organizations. Any ambiguity in classification was resolved by consensus among 5 reviewers to minimize misclassification bias.

All videos were coded using a standardized data extraction form developed for this study. The form included fields for creator type, disease, category, engagement metrics, and the quality assessment instruments (GQS and mDISCERN), allowing reviewers to score each video within the same structured framework. Five reviewers independently coded all videos, with each reviewer assessing approximately 20% of the total sample and at least 2 reviewers overlapping on every video to ensure consistency. Coding discrepancies were reviewed collectively after initial scoring and resolved by consensus at the end of data collection to ensure consistency across raters. For videos that claimed to cite peer-reviewed literature or clinical guidelines, reviewers verified the presence of such sources through in-video text, captions, or linked resources. If verification was not possible, the citation claim was coded as absent.

Video quality was assessed using 2 established tools: the GQS and the mDISCERN instrument. The GQS, originally developed for patient-facing websites and adapted for short-form videos, rates content on a 5-point Likert scale (1=poor, 5=excellent) based on flow, educational value, and comprehensiveness (Table 1) [20]. The mDISCERN, adapted from the original DISCERN framework for written materials, evaluates reliability, balance, and overall quality (Table 2) [21]. The full mDISCERN tool can be found in Multimedia Appendix 1.

**Table 1.** Global Quality Scale scoring framework.

Likert scale score	Quality assessment of the content	Information coverage	Patient utility
1	Poor quality	None	Very unlikely to be of any use to patients
2	Poor quality	Some information present	Of very limited use to patients
3	Suboptimal flow	Some information covered but important topics are missing	Somewhat useful to patients
4	Good quality and flow	Most important topics covered	Useful to patients
5	Excellent quality and flow	Most important topics covered	Highly useful to patients

**Table 2.** Modified DISCERN tool description.

Category	Example question	Explanation
Reliability of content	Are the aims of the video clear?	Does the creator state the purpose (eg, educate, raise awareness, and share experience)?
Quality of treatment information	Does the video discuss multiple perspectives?	If treatments are mentioned, are pros, cons, and alternatives presented?
Overall rating	Rate the overall quality of the video	Consider all criteria above to provide a holistic score

Both instruments have been successfully applied in prior analyses of TikTok health content [12,17]. These instruments were selected because they provide standardized, validated frameworks for assessing health information quality, which allow cross-study comparisons despite their limitations in capturing certain stylistic features of short-form videos, such as pacing and audiovisual emphasis. To address these limitations, we applied the mDISCERN instrument, which has been adapted for short-form and audiovisual health content in prior TikTok-based research [18] and used alongside GQS in several video-based quality evaluations [17,19], and conducted reviewer calibration to ensure consistent scoring. Prior to data collection, all reviewers completed calibration exercises using sample videos to ensure consistent scoring. Any discrepancies in scoring were resolved through consensus discussion among the 5 reviewers. Table 2 was developed for this study using criteria derived from the original DISCERN instrument.

Descriptive statistics summarized the distribution of videos across creator types and diseases. GQS and mDISCERN scores were compared across creator types using Kruskal-Wallis tests due to nonparametric score distributions, followed by Dunn post hoc tests for pairwise comparisons. Chi-square tests examined associations between creator type and the citation of peer-reviewed literature or clinical guidelines. All analyses were conducted using SPSS (version 28.0; IBM Corp), with significance set at *P*<.05.

Engagement metrics, including views, likes, shares, and comments, were recorded. To adjust for variability in posting time, a normalized engagement ratio (NER; interactions per 1000 views) was calculated using the formula:


$$\text{NER}=\frac{\text{likes}+\text{comments}+\text{shares}}{\text{views}/1000}$$

This allowed for standardized comparisons of audience interaction, as supported by prior TikTok engagement studies [15,19]. A higher NER reflects proportionally greater viewer interaction relative to exposure; however, it does not necessarily indicate the quality or meaningfulness of that engagement.

### Ethical Considerations

This study analyzed publicly available TikTok videos related to rare genetic conditions. All data were deidentified and obtained from open-access sources that do not require an account or login for access. In accordance with the determination provided by the Sam Houston State University Institutional Review Board (IRB), the project was classified as not human subjects research under 45 CFR 46.102. Therefore, formal IRB review was not required. The IRB confirmed that this classification applied because no identifiable private information or interaction with human participants occurred.

## Results

**Figure 1.**
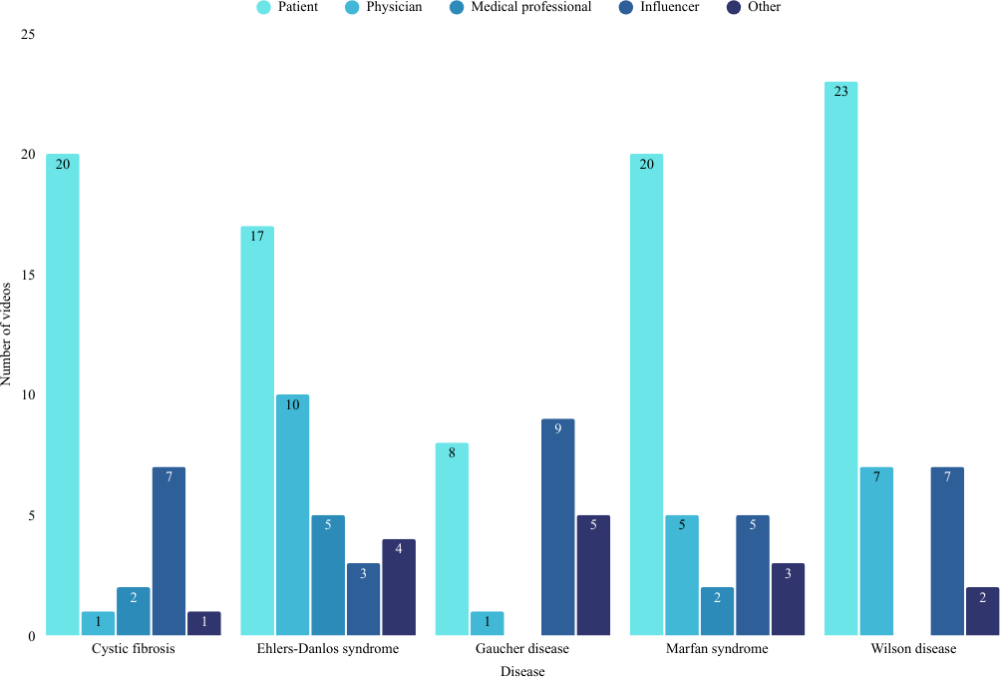
Distribution of TikTok videos by creator type and disease category.

The distribution of videos by disease and creator type is summarized in Figure 1. Patient-created content consistently represented the largest proportion across most diseases. Collectively, 184 videos amassed more than 123 million views and 9.6 million likes. Influencer-generated content accounted for the highest total views (60,992,870/132,058,986, 46.2%), followed by patient-generated videos (45,469,801/132,058,986, 34.4%) and physician-generated videos (19,000,622/132,058,986, 14.4%). Medical professional– and nonprofit organization–generated content contributed smaller proportions of total views (2,413,400/132,058,986, 1.8% and 2,964,198/132,058,986, 2.3%, respectively), with other creator types accounting for the remaining 0.9% (1,218,095/132,058,986) of views (Table 3).

**Table 3.** Summary of TikTok engagement metrics and normalized engagement ratios (NER) by creator type (N=188).

Creator type	Average views (SD)	Average likes (SD)	Total views (N=132,058,986), n (%)	Total likes and shares (N=9,836,670), n (%)	NER (per 1000 views)
Physician	791,693 (1,310,843)	25,851 (41,535)	19,000,622 (14.4)	704,288 (7.2)	2433.50
Patient	516,702 (1,489,930)	37,997 (127,974)	45,469,801 (34.4)	3,428,069 (34.9)	48.8
Influencer	1,967,512 (8,715,477)	174,542 (896,043)	60,992,870 (46.2)	5,455,019 (55.4)	30.2
Medical professional	268,156 (390,437)	12,975 (27,601)	2,413,400 (1.8)	128,585 (1.3)	32.9
Nonprofit organization	71,653 (157,967)	2587 (7034)	2,964,198 (2.3)	72,740 (0.7)	29.2
Other	197,613 (483,735)	4624 (7713)	1,218,095 (0.9)	47,969 (0.5)	28.3

While influencers and patients generated the highest overall engagement, with 60.9 million (46.2%) and 45.5 million (34.4%) views, respectively, physician-created videos had the highest NER, averaging 2433 interactions per 1000 views. This was substantially higher than the ratios for medical professionals (48.8), influencers (30.2), patients (32.9), nonprofit organizations (29.2), and other creators (28.3). The NER adjusts for differences in video reach, providing a clearer measure of content’s interactive quality rather than just popularity. Full results are shown in Table 3.

Content produced by physicians and medical professionals demonstrated significantly higher quality and reliability, as measured by the GQS and the mDISCERN. Physician-created videos had a mean GQS score of 3.62 (SD 0.71) and mDISCERN score of 3.21 (SD 0.65), while videos from medical professionals scored slightly higher (GQS mean 3.89, SD 0.66; mDISCERN mean 3.11, SD 0.58). In contrast, videos from influencers and patients exhibited notably lower-quality scores (influencers: GQS mean 1.48, SD 0.6; mDISCERN mean 1.4, SD 0.55; patients: GQS mean 1.57, SD 0.58; mDISCERN mean 1.38, SD 0.52; Table 4). A full breakdown of consensus data is available in Multimedia Appendix 2.

**Table 4.** Average Global Quality Scale (GQS) and modified DISCERN (mDISCERN) scores by creator type.

Creator type	GQS score, mean (SD)	mDISCERN score, mean (SD)
Physician	3.62 (0.71)	3.21 (0.65)
Medical professional	3.89 (0.66)	3.11 (0.58)
Influencer	1.48 (0.60)	1.42 (0.55)
Patient	1.57 (0.58)	1.38 (0.52)
Nonprofit organization	2.92 (0.70)	2.60 (0.66)
Other	2.73 (0.68)	2.48 (0.61)

For EDS (40/184, 21.7%), Kruskal-Wallis tests revealed significant variation in GQS scores (H=24.28; *P*<.001; ε²=0.57, 95% CI 0.41‐0.72) and in mDISCERN scores (H=24.09; *P*<.001; ε²=0.56, 95% CI 0.39‐0.71). For Wilson disease (40/184, 21.7%), significant differences were found in GQS (H=30.50; *P*<.001; ε²=0.75, 95% CI 0.60‐0.84) and mDISCERN (H=29.89; *P*<.001; ε²=0.73, 95% CI 0.58‐0.82). For cystic fibrosis (34/184, 18.5%), significant variation was observed in GQS (H=12.36; *P*=.03; ε²=0.26, 95% CI 0.04‐0.47) and mDISCERN (H=11.96; *P*=.04; ε²=0.25, 95% CI 0.03‐0.45). No significant differences in quality scores were found for Marfan syndrome (GQS: H=4.87, *P*=.43; mDISCERN: H=7.04, *P*=.22) or Gaucher disease (GQS: H=8.59, *P*=.07; mDISCERN: H=5.46, *P*=.24; Table 5). No statistically significant differences in quality scores were found among creator types for Marfan syndrome (GQS: H=4.87, *P*=.43; mDISCERN: H=7.04, *P*=.22) or Gaucher disease (GQS: H=8.59, *P*=.07; mDISCERN: H=5.46, *P*=.24). The lack of significance may be attributed to smaller sample sizes and limited variation in creator type representation.

**Table 5.** Statistical analysis of information quality scores by content creator type for each rare genetic disease.

Disease	Highest-scoring creator types	Global Quality Scale score			Modified DISCERN score		
		H	*P* value	ε² (95% CI)	H	*P* value	ε² (95% CI)
Ehlers-Danlos syndrome	Medical professionals and physicians	24.28	<.001	0.57 (0.41-0.72)	24.09	<.001	0.56 (0.39-0.71)
Wilson disease	Medical professionals and physicians	30.50	<.001	0.75 (0.60-0.84)	29.89	<.001	0.73 (0.58-0.82)
Cystic fibrosis	Medical professionals and physicians	12.36	.03	0.26 (0.04-0.47)	11.96	.04	0.25 (0.03-0.45)
Marfan syndrome	Nonprofit organizations and others	4.87	.43	0 (0-0.08)	7.04	.22	0.06 (0-0.13)
Gaucher disease	Physicians and patients	8.59	.07	0.15 (0.02-0.29)	5.46	.24	0.09 (0-0.22)

Dunn test with Bonferroni correction revealed that physician-generated videos scored significantly higher on the GQS than both influencer videos (adjusted *P*=.004) and patient-created videos (adjusted *P*=.007). Likewise, content from other medical professionals outperformed influencer videos on GQS (adjusted *P*=.01). On mDISCERN, physician videos again exceeded influencer (*P*=.003) and patient videos (*P*=.005), and other content exceeded influencer videos (*P*=.02). No other pairwise contrasts reached significance. Full pairwise comparison results are available in Multimedia Appendix 3.

**Figure 2.**
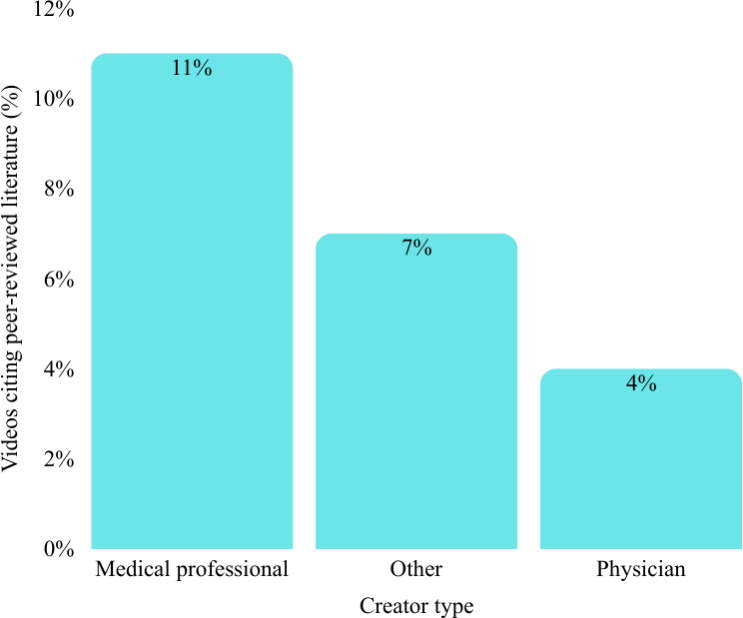
Proportion of TikTok videos citing peer-reviewed references by creator type.

Chi-square analysis of the proportion of videos citing peer-reviewed literature or official guidelines showed no statistically significant differences among creator types (*χ*²_5_=10.6; *P*=.07). Overall, only 7 (3.8%) videos included scientific references. Videos created by medical professionals had the highest citation rate (3/28, 11%), followed by those categorized as by others (1/15, 7%) and by physicians (3/88, 4%). Despite these relative differences, the consistently low citation rates across all creator groups highlight a general lack of evidence-based referencing in TikTok content related to rare diseases (Figure 2).

## Discussion

### Principal Findings

This study highlights the growing importance of TikTok as a platform for rare disease awareness and education, while also emphasizing critical disparities in the quality of medical information shared. Most videos analyzed were created by patients and influencers, who collectively attracted the highest levels of viewer engagement. Despite this popularity, these videos generally contained lower-quality medical information compared with content produced by medical professionals and physicians. This pattern aligns with previous research across other health conditions, where content created by medical professionals typically receives less audience engagement despite higher-quality scores [14,16]. Although physician-generated videos represented fewer total uploads and reached smaller audiences overall, they demonstrated higher NER compared with those produced by nonphysicians, suggesting that audience interaction may be influenced by perceived credibility or informational clarity rather than entertainment value, reflecting prior TikTok research showing a mismatch between content popularity and quality [11,13].

This study found substantial differences in quality scores across creator types. Physician- and medical professional–generated videos consistently demonstrated higher quality based on the GQS and the mDISCERN. Conversely, influencer-created and patient-generated videos scored notably lower on these scales. These findings mirror results from previous TikTok evaluations of various health topics, including diabetes, fibromyalgia, and coronary artery disease, where professionally created content consistently demonstrated greater accuracy but attracted less viewer attention [11-13].

An important observation of this analysis was the pronounced mismatch between content quality and viewer engagement. Physician- and medical professional–generated videos, despite higher quality, received fewer views and interactions compared with influencer- or patient-generated content. Influencer-created videos, in particular, garnered substantial viewership despite notably lower-quality ratings. This discrepancy, previously documented in studies on sinusitis and eating disorders on TikTok, raises significant concerns regarding public health literacy and underscores the risks associated with widespread misinformation on short-form video platforms [13,16]. The NER further emphasizes this mismatch, demonstrating that high-quality physician-created videos, despite fewer total views, generated significantly higher engagement per view. This suggests that viewers might interact more meaningfully with accurate, high-quality content even when encountered less frequently.

In the context of rare diseases, this disconnect between popularity and accuracy is particularly problematic. Patients frequently experience prolonged diagnostic delays, inconsistent expertise of health care professionals, and fragmented health care management [1,5]. Therefore, many individuals rely on platforms like TikTok for disease-specific information, emotional support, and practical management strategies [4,8]. This trend aligns with broader findings within rare disease communities, where patients and caregivers commonly use digital tools to access reliable information, reduce isolation, and navigate fragmented health care systems [22]. Foster and Ellis [23] suggest that self-diagnosis behaviors driven by platforms like TikTok may reflect complex motivations, including identity exploration, community seeking, and barriers to formal health care access. However, while patient-generated videos offer considerable emotional and community building value, they often include medically inaccurate, incomplete, or misleading information, reinforcing concerns highlighted by prior research [12,15].

Another critical finding was the notably low rate of citing peer-reviewed literature or clinical guidelines across all types of creators. Although medical professionals and physicians cited evidence slightly more frequently than influencers or patients, overall citation rates remained exceptionally low. This aligns with previous analyses of TikTok content in osteoporosis, cancer, and gastrointestinal health, where minimal referencing of credible sources was also noted [17,19]. The absence of evidence-based citations severely limits the educational effectiveness of these videos, hindering viewers’ ability to evaluate content credibility and posing tangible risks to patient safety, particularly within vulnerable rare disease populations [6].

Despite these challenges, TikTok presents significant opportunities for community support, increased visibility, and patient empowerment within rare disease communities. Patient-generated content can effectively raise awareness, provide emotional support, and amplify visibility for conditions often overlooked in mainstream health care and research [2,9]. Prior research demonstrates that hashtags like #RareDisease and #ChronicIllness serve as valuable online gathering spaces, facilitating supportive communities and collective advocacy [8,24]. For individuals living with rare conditions, often marked by isolation and limited support, sharing personal experiences can foster identity formation, validation, and belonging. Patient-created videos may help reduce stigma, increase awareness among peers, and promote self-advocacy, even when medical accuracy is imperfect. These peer-to-peer interactions complement rather than replace professional education and highlight the platform’s potential as a space for empowerment and community building.

To effectively leverage TikTok’s potential while addressing content accuracy and reliability, a multifaceted approach is required. Institutional support, such as training health care professionals in digital health communication, actively involving patient advocates in content creation, and implementing platform-level enhancements, including verified creator badges and algorithms prioritizing credible, evidence-based content, could significantly improve the quality and reliability of shared information [25-27]. Additionally, enhancing digital health literacy through targeted educational initiatives can empower viewers to critically evaluate online health information, fostering safer online communication practices [3,28].

In addition to institutional and platform-level interventions, collaboration between health care professionals and patient influencers may represent a promising strategy for improving the quality and reach of health information. Training programs that equip patients and influencers with foundational health communication and media literacy skills could strengthen the accuracy and clarity of their content while preserving authenticity. Likewise, structured partnerships between physicians, allied health professionals, and experienced content creators could expand credible outreach and help translate complex medical concepts into accessible, patient-centered narratives. Within rare disease communities, such training initiatives could be developed in collaboration with established advocacy organizations such as the National Organization for Rare Disorders, which already supports educational outreach and patient empowerment programs. Such approaches may ultimately enhance trust, reduce misinformation, and promote more equitable participation in digital health education.

While these strategies may offer promising directions for improving content quality and engagement on social media platforms, several limitations of this study should be acknowledged. While EDS, Wilson’s disease, and cystic fibrosis showed significant variation in information quality across creator types, results for Marfan and Gaucher diseases were not statistically significant. This likely reflects smaller sample sizes and limited creator diversity within these subgroups, which reduced statistical power to detect true differences.

The cross-sectional design captures only a single snapshot in TikTok’s rapidly evolving content environment, potentially limiting generalizability. The reliance on hashtags and English-language videos may have excluded relevant content, highlighting the need for broader multilingual research approaches. Additionally, the inclusion of only the most viewed videos introduces potential selection bias, as this approach may overrepresent popular content while underrepresenting less visible yet potentially higher-quality educational videos. The GQS and mDISCERN tools, though validated, were originally developed for longer-form content, underscoring the need for specialized assessment tools tailored specifically to short-form videos [29]. It is possible that certain diseases were more likely to include peer-reviewed citations than others, reflecting differences in research visibility, professional engagement, or the maturity of patient advocacy communities; however, this relationship was not specifically examined in this analysis. Future research using longitudinal designs could provide deeper insights into the direct impact of social media content on health behaviors and outcomes, further elucidating the platform’s long-term educational efficacy.

### Conclusions

TikTok has become a significant platform for health communication, especially for rare disease communities, facilitating patient empowerment, advocacy, and education. However, our findings highlight substantial gaps in the quality and reliability of TikTok content on rare genetic conditions. Videos from patients and influencers are highly popular yet frequently lack medical accuracy and proper citations. In contrast, physicians and medical professionals produce consistently higher-quality content that remains comparatively underviewed.

Although physician-generated content had significantly higher-quality scores, influencer- and patient-generated videos dominated views. This discrepancy raises concerns about misinformation and its implications for patient safety, consistent with prior findings. Institutional support, such as targeted training in digital health communication, could empower health care professionals to produce engaging, evidence-based content. Addressing this imbalance requires increased collaboration between clinicians and patient creators, institutional training in digital health communication, and targeted platform-level interventions such as verified badges and enhanced algorithmic visibility for credible content. Additionally, improving digital health literacy is essential to equip users to critically evaluate health information online. Such coordinated efforts can transform TikTok into a more reliable and effective tool for rare disease education and community support.

## Supplementary material

10.2196/79978 Multimedia Appendix 1 Pairwise Dunn post hoc comparisons of TikTok video quality by creator type: *z* statistics with 2-tailed asymptotic *P* values and Bonferroni-adjusted *P* values for Global Quality Scale and modified DISCERN scores.

10.2196/79978 Multimedia Appendix 2 Modified DISCERN scoring system for TikTok.

10.2196/79978 Multimedia Appendix 3 Descriptive overview of TikTok videos by creator type and disease category. Video counts, mean Global Quality Scale and modified DISCERN scores, total views, likes, shares, and number of videos citing peer-reviewed references.

## Abbreviations

ADHD: attention-deficit/hyperactivity disorder
EDS: Ehlers-Danlos syndrome
GQS: Global Quality Scale
IRB: institutional review board
mDISCERN: modified DISCERN
NER: normalized engagement ratio
