# Supplementary material for: TikTok as a Platform for Patient Education and Health Information in Rare Genetic Diseases: Cross-Sectional Study
Source: JMIR Form Res. 2026 Feb 24;10:e79978. doi: 10.2196/79978 (PMC12931836; doi:10.2196/79978)
Supplement: Multimedia Appendix 1 [file formative-v10-e79978-s001.doc]

| **Creator Type** | **Physician** | **Patient** | **Influencer** | **Medical Prof.** | **Organization** | **Other** |
| --- | --- | --- | --- | --- | --- | --- |
| **Physician** | — | 3.42; **.0006 / .007** | **3.86; .0001 / .004** | 0.46; .647 / .650 | 0.71; .478 / .480 | 1.22; .222 / .220 |
| **Patient** |  | — | 0.41; .682 / .680 | 0.95; .342 / .340 | 0.64; .523 / .520 | 0.30; .763 / .770 |
| **Influencer** |  |  | — | **3.29; .0010 / .010** | 1.10; .271 / .270 | 1.57; .116 / .120 |
| **Medical Prof.** |  |  |  | — | 0.55; .580 / .580 | 0.76; .448 / .440 |
| **Organization** |  |  |  |  | — | 1.39; .164 / .160 |
| **Other** |  |  |  |  |  | — |
