# Supplementary material for: TikTok as a Platform for Patient Education and Health Information in Rare Genetic Diseases: Cross-Sectional Study
Source: JMIR Form Res. 2026 Feb 24;10:e79978. doi: 10.2196/79978 (PMC12931836; doi:10.2196/79978)
Supplement: Multimedia Appendix 2 [file formative-v10-e79978-s002.docx]

Instructions: Score each video on a scale from 1 (No) to 5 (Yes), based on the criteria below.

Consider the short-form nature of TikTok videos when scoring. 
 
**Section 1: Reliability of the Content** 
**Are the aims of the video clear?** 
Does the creator clearly state the purpose of the video (e.g., to educate, raise awareness, share personal experience)? 

**Does the video achieve its aims?**

Does the content deliver on its stated purpose effectively? 

**Is the video relevant to its intended audience?** 
Does it provide information that is understandable and useful for viewers seeking knowledge about the condition?

**Is the information supported by credible sources?** 
Does the video mention peer-reviewed research, guidelines, or reputable organizations (e.g., links, overlays, captions)?

**Are the sources of information transparent?** 
Does the creator mention where their information comes from (e.g., medical articles, personal experience, professional knowledge)? 

**Is the information current?** 
Is the content consistent with current medical understanding, guidelines, or updates? 

**Does the video avoid misleading or biased information?** 
Is the content free of exaggerated claims, clickbait language, or unverified advice?

**Section 2: Quality of Treatment Information** 
**Does the video discuss multiple perspectives or treatment options?** 
If treatments are mentioned, does it provide a balanced overview (e.g., pros, cons, alternatives)? 

**Does the video address uncertainties or limitations?** 
Are potential risks, unanswered questions, or limitations of the information presented? 

**Does the video provide credible resources for further information?** 
Are viewers directed to reliable sources for additional reading or support (e.g., links in the description, external resources)? 

**Overall Rating** 
Rate the overall quality of the video as a source of health information. 
Consider all criteria above to provide a holistic score.
