# Supplementary material for: TikTok as a Platform for Patient Education and Health Information in Rare Genetic Diseases: Cross-Sectional Study
Source: JMIR Form Res. 2026 Feb 24;10:e79978. doi: 10.2196/79978 (PMC12931836; doi:10.2196/79978)
Supplement: Multimedia Appendix 3 [file formative-v10-e79978-s003.doc]

| **Ehlers-Danlos** | | | | | | | |
| --- | --- | --- | --- | --- | --- | --- | --- |
| **Creator Type** | **Video Count** | **Mean GQS** | **Mean mDISCERN** | **Total Views** | **Total Likes** | **Total Shares** | **Peer Reviewed Reference Count** |
| **Influencer** | 3 | 1.66 | 1.66 | 3205677 | 171164 | 6969 | 0 |
| **Medical Professional** | 5 | 4.40 | 3.40 | 2154600 | 110352 | 11524 | 1 |
| **Organization** | 1 | 5.00 | 3.00 | 108600 | 4993 | 3514 | 0 |
| **Other** | 4 | 2.50 | 2.00 | 611000 | 43053 | 3051 | 1 |
| **Patient** | 17 | 1.88 | 1.529 | 12106500 | 668236 | 65563 | 0 |
| **Physician** | 10 | 3.70 | 3.60 | 12022352 | 501837 | 80357 | 0 |
| **Marfan Syndrome** | | | | | | | |
| **Creator Type** | **Video Count** | **Mean GQS** | **Mean mDISCERN** | **Total Views** | **Total Likes** | **Total Shares** | **Peer Reviewed Reference Count** |
| **Influencer** | 5 | 1.40 | 1.00 | 2681418 | 56198 | 8084 | 0 |
| **Medical Professional** | 2 | 2.50 | 2.00 | 83300 | 1126 | 227 | 0 |
| **Organization** | 5 | 3.00 | 2.40 | 710440 | 30054 | 223 | 0 |
| **Other** | 3 | 3.00 | 2.66 | 2044061 | 17579 | 259 | 0 |
| **Patient** | 20 | 1.75 | 1.25 | 6135140 | 313415 | 10656 | 0 |
| **Physician** | 5 | 2.00 | 1.60 | 4073700 | 31393 | 1581 | 0 |
| **Wilson's Disease** | | | | | | | |
| **Creator Type** | **Video Count** | **Mean GQS** | **Mean mDISCERN** | **Total Views** | **Total Likes** | **Total Shares** | **Peer Reviewed Reference Count** |
| **Influencer** | 7 | 1.71 | 1.42 | 81796 | 635 | 61 | 0 |
| **Medical Professional** | 0 | 0.00 | 0.00 |  |  |  | 0 |
| **Organization** | 1 | 5.00 | 4.00 | 184 | 4 | 0 | 0 |
| **Other** | 2 | 2.50 | 2.00 | 255461 | 7979 | 34 | 0 |
| **Patient** | 23 | 1.04 | 1.04 | 639665 | 7998 | 208 | 0 |
| **Physician** | 7 | 5.00 | 3.85 | 502537 | 22926 | 1313 | 0 |
| **Cystic Fibrosis** | | | | | | | |
| **Creator Type** | **Video Count** | **Mean GQS** | **Mean mDISCERN** | **Total Views** | **Total Likes** | **Total Shares** | **Peer Reviewed Reference Count** |
| **Influencer** | 7 | 1.28 | 1.57 | 53772400 | 5143709 | 30406 | 0 |
| **Medical Professional** | 2 | 4.00 | 3.50 | 175500 | 5297 | 59 | 0 |
| **Organization** | 3 | 2.33 | 2.66 | 226854 | 6792 | 208 | 0 |
| **Other** | 1 | 2.00 | 2.00 | 48200 | 680 | 28 | 0 |
| **Patient** | 20 | 1.35 | 1.40 | 23247725 | 1781564 | 8642 | 0 |
| **Physician** | 1 | 3.00 | 3.00 | 2400000 | 64200 | 619 | 0 |
| **Gaucher Disease** | | | | | | | |
| **Creator Type** | **Video Count** | **Mean GQS** | **Mean mDISCERN** | **Total Views** | **Total Likes** | **Total Shares** | **Peer Reviewed Reference Count** |
| **Influencer** | 9 | 1.44 | 1.44 | 1251579 | 39089 | 704 | 0 |
| **Medical Professional** | 0 | 0.00 | 0.00 |  |  |  | 0 |
| **Organization** | 7 | 2.57 | 2.42 | 172017 | 2136 | 45 | 0 |
| **Other** | 5 | 1.80 | 1.60 | 5476 | 64 | 13 | 0 |
| **Patient** | 8 | 2.50 | 2.25 | 3340771 | 572511 | 2276 | 0 |
| **Physician** | 1 | 4.00 | 4.00 | 2033 | 59 | 3 | 1 |
